# Role of echocardiography in CRT

**DOI:** 10.18632/aging.101687

**Published:** 2018-11-26

**Authors:** Bhupendar Tayal, Peter Sogaard

**Affiliations:** 1 Aalborg University Hospital, Department of Cardiology, Aalborg, Denmark

**Keywords:** **Keywords: **heart failure, cardiac resynchronization therapy, echocardiography, dyssynchrony, activation delay

Cardiac resynchronization therapy (CRT) is an effective treatment for patients with heart failure (HF) and conduction abnormalities. Conceptually, CRT works by improving myocardial contraction synchrony and performance with a parallel left ventricular (LV) reverse remodeling and subsequent improvement in morbidity and mortality [1-3]. Current CRT guidelines include electrical dyssynchrony reflected by widening of QRS (>150ms) and left bundle branch block (LBBB) QRS morphology as a class I indication. Despite improvement, ECG criteria for selecting patients are not perfect and nearly one-third of patients do not respond to CRT.

CRT mechanically ameliorates the activation delay between opposing contracting walls of LV by an extra lead implanted into LV free wall. Echocardiography can provide the additional necessary improvement in selection of patients by identifying more precisely the asymmetry in contraction and activation delay between the opposing contracting walls of LV. Over the years, a number of echocardiographic techniques have been introduced to identify this asymmetry more commonly known as mechanical dyssynchrony [4]. Despite showing promising role, echocardiographic dyssynchrony has never been implemented in the guidelines. It is because most of the studies having mechanical dyssynchrony as one of the selection criteria applied time-to-peak method of dyssynchrony assessment and therefore failed in multicenter settings [5, 6]. Time-to-peak dyssynchrony can occur in patients with HF due to fibrosis or reduced contractility and do not necessarily represents a true activation delay or in other words a substrate to CRT response [7].

Recent studies applied some new echocardiographic techniques focusing on the physiology of a how a CRT works which have shown promising results in small-scale studies [1, 3]. One among them is cross correlation analysis (CCA) where activation delay between opposing contracting walls of LV can be calculated. Presence of a significant activation delay by this method prior to device implantation was found to be associated with improved outcome in patients with wide QRS (≥ 120ms) [2]. Moreover, applying this method it was demonstrated that CRT can be hazardous to patients specifically if implanted in those without activation delay at baseline 1. Further applying echocardiography a contraction pattern can be identified particularly among patients with LBBB and lack of this contraction pattern prior to CRT implantation at baseline is found to be associated with poor prognosis [3].

This editorial focusses on the sub-study performed on the Echocardiography guided cardiac resynchronization therapy (EchoCRT) trial data by Tayal et al. [8]. In EchoCRT trial HF patients with narrow QRS (< 130 ms) and dyssynchrony by echocardiography were randomized to CRT-On and CRT-off [6]. The trial demonstrated that CRT do not benefit patients with narrow QRS. Furthermore, higher mortality was observed among patients randomized to CRT-On. One of the criteria for inclusion was the presence of mechanical dyssynchrony by echocardiography. Time-to-peak based methods of dyssynchrony were applied either by radial strain delay (> 130 ms) or tissue Doppler imaging (TDI) opposing wall delay (> 80ms). In the sub-study by Tayal et al. [8], CCA method was applied to ascertain activation delay among the patients included in the EchoCRT trial. By applying this method, it was observed that nearly 50% had no activation delay despite presence of time-to-peak dyssynchrony. The major finding of the study was that patients with lack of activation delay by CCA had poor outcome if randomized to CRT-On which was most like due to the risk of device induced activation delay. There was nearly 1.7 times increased risk of HF hospitalization or death among patients with lack of activation delay at baseline randomized to CRT-On.

This study highlights the limitation of time-to-peak dyssynchrony methods, bringing forward the discussion on the role of dyssynchrony in patients treated with CRT and its physiology. Future trials applying these newer echocardiographic techniques in selection of patients with wide QRS (> 130ms) for CRT in multicenter randomized settings can provide more convincing evidence. Specifically focusing on groups where the guidelines are still ambiguous, such as LBBB patients with intermediate QRS duration (130-149 ms) and non-LBBB morphology (> 130ms) would be of benefit by reducing unnecessary implants.
